# Protocol for immunomagnetic enrichment of CD8 T cells from complex murine tissues

**DOI:** 10.1016/j.xpro.2026.104456

**Published:** 2026-04-02

**Authors:** Ella Trolio, Lelinh Duong, Michaela Lucas, Amy Prosser

**Affiliations:** 1 Medical School, University of Western Australia, Perth, WA 6009, Australia; 2 Department of Immunology, Sir Charles Gairdner Hospital and PathWest Laboratory Medicine, Perth, WA 6009, Australia

**Keywords:** Cell isolation, Cell separation/fractionation, Flow Cytometry, Immunology, Model Organisms

## Abstract

Efficient isolation of CD8 T cells is essential for accurately investigating their roles in immune regulation and disease pathogenesis. Here, we present a protocol for the isolation and enrichment of CD8 T cells by positive selection from complex murine tissues, including bone marrow (BM), liver, heart, and kidneys. We describe steps for tissue harvesting and processing into single-cell suspensions. We then detail procedures for immunomagnetic CD8 T cell enrichment and assessment of cell purity and viability by flow cytometry.

## Highlights

• Isolation of leukocytes from murine BM, liver, heart, and kidneys
• Non-enzymatic dissociation of kidney and heart tissue
• Protocol for immunomagnetic enrichment of CD8 T cells
• Flow cytometry analysis of CD8 T cell purity and viability

## Abstract (editor highlights)

Publisher’s note: Undertaking any experimental protocol requires adherence to local institutional guidelines for laboratory safety and ethics.

## Graphical abstract

**Figure:**
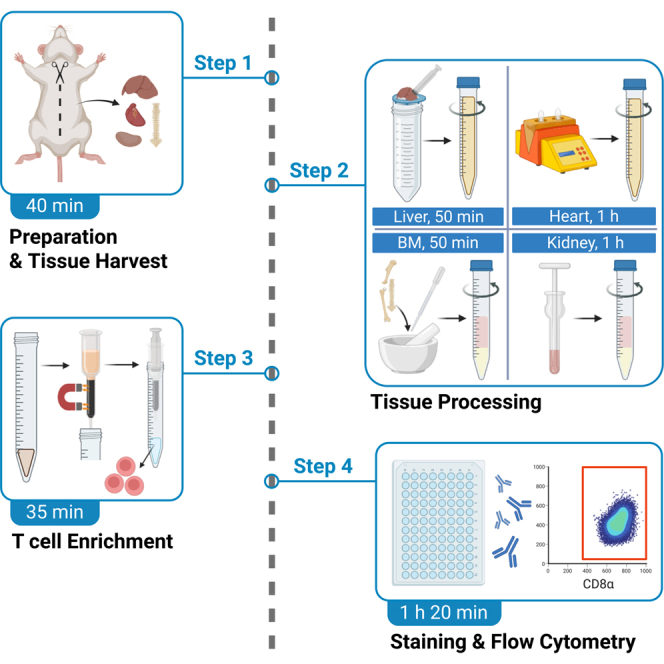

## Before you begin

The following protocol describes the isolation and enrichment of mouse CD8 T cells by immunomagnetic positive selection. This protocol is specific to the enrichment of CD8 T cells. Substitution with other commercial kits to enrich alternative leukocyte subsets would require validation. Tissue processing steps have been individually optimized for each organ to maximize tissue-specific enrichment efficiency. For CD8 T cell enrichment from spleen and lymph node tissue, adherence to the manufacturer’s standard instructions is generally sufficient.

The time specified for the enrichment step is for a single sample. Several samples can be enriched at once using multiple magnets, though staggered incubation times are recommended. Preparation of required reagents and materials in advance is essential to minimize cell death and maximize yield.

### Innovation

Isolating specific immune cell populations from mouse tissues is essential for many downstream applications. While fluorescence activated cell sorting achieves high purity isolation, it is often time consuming for complex tissues and can induce cell stress due to high pressure fluidics. Magnetic separation offers a faster alternative that reduces cell stress, but standard kits are generally optimized for common tissues such as blood, spleen and lymph nodes. Here, we present an immunomagnetic method for CD8 T cell enrichment specifically optimized for complex tissues, where high purity magnetic separation has not previously been described. We also describe a non-enzymatic method for processing heart and kidney tissue, making this protocol compatible with downstream applications such as proteomic analysis, where enzymatic cleavage could compromise results.

### Institutional permissions

All experiments conducted to establish this protocol were approved by the Harry Perkins Institute of Medical Research Animal Ethics Committee (protocols AE173, AE279 and AE372) and adhered to the Australian Code for the Care and Use of Animals for Scientific Purposes. Appropriate animal ethics approval must be obtained in accordance with institutional and national guidelines and regulations.

### Prepare required reagents

**Timing: 30 min**

1. Prepare the following reagents as outlined in the materials and equipment section:
  a. FACS buffer (PBS, pH 7.4 with 2% heat-inactivated newborn calf serum (HI-NCS)).
  b. MACS buffer (PBS, pH 7.2 with 0.5% bovine serum albumin (BSA) and 2 mM EDTA).
  c. DMEM (high glucose + glutamine + pyruvate) with 10% HI-NCS.
  d. 42% isotonic Percoll.
  e. 44% isotonic Percoll.
  f. 70% isotonic Percoll.
  g. Red blood cell (RBC) lysis solution.
  h. Zombie UV viability dye.
  i. Surface antibody cocktail.
  j. BD Stabilizing Fixative.

### Prepare for tissue harvest

**Timing: 10 min**

2. Collect the following equipment:
  a. Anesthetic, 70% ethanol, dissection board, surgical tape, surgical scissors, straight forceps, clamp forceps, cold saline (sodium chloride 0.9% for irrigation), cotton tip applicators, cotton balls, ice, bags for waste and carcass disposal.
3. Prepare the following equipment and reagents per mouse:
  a. 3 mL syringe with 30 G needle filled with 3 mL cold saline.
  b. Liver harvest.
    i. 1 × 5 mL tube filled with 4mL DMEM.
  c. Heart harvest.
    i. 1 × 5 mL tube filled with 4mL DMEM + 10% HI-NCS.
  d. Kidney harvest.
    i. 1 × 5 mL tube filled with 4mL DMEM + 10% HI-NCS.
  e. BM harvest.
    i. 3 × 5 mL tubes filled with 4mL DMEM.

## Key resources table

**Table:** 

REAGENT or RESOURCE	SOURCE	IDENTIFIER
**Antibodies**		

TCRβ AlexaFluor 700 (H57-597) 1:50	BioLegend	Cat#109223
CD11b Brilliant UV 395 (M1/70) 1:200	BD Biosciences	Cat#563553
CD8β Brilliant Violet 480 (H35–17.2) 1:800	BD Biosciences	Cat#746835
CD4 Super Bright 600 (OX35) 1:300	Thermo Fisher	Cat#63-0042-80
CD8α Super Bright (53–6.7) 645 1:200	Thermo Fisher	Cat#64-0081-82
CD45.2 APC/eFluor 780 (104) 1:200	Thermo Fisher	Cat#47-0454-82
CD45R/B220 Alexa Fluor 594 (RA3-6B2) 1:400	BioLegend	Cat#103254
F4/80 AlexaFluor 647 (T45-2342) 1:100	BD Biosciences	Cat#565853
CD335/NKp46 Brilliant UV 737 (29A1.4) 1:100	BD Biosciences	Cat#612805
Ly6G FITC (1A8) 1:100	BD Biosciences	Cat#551460

**Chemicals, peptides, and recombinant proteins**		

DMEM (with high glucose + glutamine + pyruvate)	Thermo Fisher	Cat#10569010
Percoll	Cytiva Life Sciences	Cat#17–0891-01
Distilled H2O for irrigation	Baxter	Cat#AHF7113
PBS tablets	Thermo Fisher	Cat#18912014
10X PBS	Thermo Fisher	Cat#70013-032
Heat-inactivated newborn calf serum (HI-NCS)	Thermo Fisher	Cat#26010-074
Red blood cell lysis solution	Tonbo	Cat#TNB-4300
MACS® BSA Stock Solution	Miltenyi Biotec	Cat#130-091-376
autoMACS® Rinsing Solution	Miltenyi Biotec	Cat#130-091-222
Sodium chloride 0.9% for irrigation	Baxter	Cat#AHF7123

**Critical commercial assays**		

Zombie UV Fixable Viability Kit	BioLegend	Cat#423107
Brilliant Stain Buffer	BD Biosciences	Cat#566349
BD Stabilizing Fixative	BD Biosciences	Cat#338036
CD8 (TIL) MicroBeads, mouse	Miltenyi Biotec	Cat#130-116-478

**Experimental models: Organisms/strains**		

Mouse BALB/c: male/female, 13-24 weeks	Animal Resources Centre (ARC, later Ozgene-ARC)	BC
Mouse C57BL/6J: male/female, 13-24 weeks	Animal Resources Centre (ARC, later Ozgene-ARC)	B6
Mouse CD45.1 BALB/c (CByJ.SJL(B6)-Ptprc^a^/J): male/female, 13-24 weeks	The Jackson Laboratory	Strain#006584
Mouse CD45.1 C57BL/6J (B6.SJL-Ptprc^a^Pepc^b^/BoyJ): male/female, 13-24 weeks	Animal Resources Centre (ARC, later Ozgene-ARC)	PTP

**Software and algorithms**		

FlowJo v10	BD Biosciences	RRID:SCR_008520: https://flowjo.com/flowjo/download

**Other**		

Isoflurane anesthetic (Isothesia)	Henry Schein	Cat#988–3244
Dissection board	DispoCut	Cat#M630-1
Surgical tape	3M	Cat#1530-0
Straight forceps	INKA Surgical Instruments	Cat#25547.15
Surgical scissors	INKA Surgical Instruments	Cat#1550.11
Clamp forceps	INKA Surgical Instruments	Cat#16305.01
Cotton balls	N/A	N/A
Cotton tip applicators	N/A	N/A
30 G Needle	BD	Cat#305106
3 mL Syringe (without needle)	Terumo	Cat#SS+03S
Scalpel (disposable, 23 blade, No. 4 handle)	Livingstone	Cat#SCP23L
Mortar and pestle	N/A	N/A
5 mL Polycarbonate tubes	Techno Plas	Cat#C5016UU
15 mL Polypropylene tubes	Thermo Fisher	Cat#188271
50 mL Polypropylene tubes	Thermo Fisher	Cat#339652
70 μm Cell strainer	Miltenyi Biotec	Cat#130-110-916
20 μm Pre-separation filter	Miltenyi Biotec	Cat#130-101-812
1 mL Transfer pipettes	Sarstedt	Cat#86.1172
60 x 15 mm petri dish	Corning	Cat#351007
MS columns	Miltenyi Biotec	Cat#130-042-201
MACS™ Multistand	Miltenyi Biotec	Cat#130-042-303
MiniMACS™ Separator	Miltenyi Biotec	Cat#130-042-102
MidiMACS™ Separator	Miltenyi Biotec	Cat#130-042-302
C Tubes	Miltenyi Biotec	Cat#130-093-237
gentleMACS™ Dissociator	Miltenyi Biotec	Cat#130-093-235
Glass dounce homogenizer	N/A	N/A
96-Well U bottom plates	Corning	Cat#353077
1.5 mL Graduated microtubes	Quality Scientific Plastics	Cat#509-GRD-Q
Countess 3 FL Automated Cell Counter	Thermo Fisher	Cat#AMQAF2000
BD LSRFortessa Flow Cytometer	BD Biosciences	N/A

## Materials and equipment

**Table:** FACS buffer

Reagent	Final concentration	Amount
1X PBS	N/A	490 mL
HI-NCS	2%	10 mL
**Total**	**N/A**	**500 mL**

Prepare in a sterile environment and store at 4°C for up to six months.

**Table:** MACS buffer

Reagent	Final concentration	Amount
MACS® BSA Stock Solution	1:20	5 mL
autoMACS® Rinsing Solution	N/A	95 mL
**Total**	**N/A**	**100 mL**

Prepare fresh on the day of use and store on ice.

***Alternatives:*** This separation buffer may also be made in-house, provided the final solution contains PBS, pH 7.2 with 0.5% BSA and 2 mM EDTA. Similar separation buffers can also be sourced from other manufacturers and may be used in this protocol after validation. For example, according to the manufacturer, EDTA can be replaced by supplements such as anticoagulant citrate dextrose formula-A or citrate phosphate dextrose, and BSA can be replaced by proteins such as mouse serum albumin, mouse serum, or fetal bovine serum. Please refer to the Alternative Resources Table for additional details.

**CRITICAL:** Buffers or media containing Ca^2+^ or Mg^2+^ are not recommended for use.

### DMEM buffer

Prepare DMEM buffer for processing heart and kidney samples by adding 10% v/v HI-NCS to the required volume of DMEM (high glucose + glutamine + pyruvate). For each mouse, ∼50mL of DMEM + 10% HI-NCS is required if both the heart and kidneys are being processed. Prepare in a sterile environment and store at 4°C for up to 4 weeks.

### Isotonic Percoll solutions

Density gradient centrifugation is a commonly used technique for isolating leukocytes from mouse tissues. Here, we use a 42% Percoll solution to separate leukocytes from parenchymal cells in the heart and liver. For more detailed information on Percoll centrifugation for heart and liver tissue, see Prosser et al. 2021.^1^ For kidneys and BM, leukocytes are isolated by overlaying a 44% Percoll solution over a 70% Percoll solution. First, 10X PBS is added to undiluted Percoll to prepare a stock isotonic solution. This isotonic Percoll is then diluted with 1X PBS or RPMI to achieve the desired working densities. RPMI is used to dilute the 44% Percoll solution to improve visual distinction between layers during overlay.

**Table:** 42% Percoll

Reagent	Final concentration	Amount
10X PBS	N/A	2.1 mL
1X PBS	N/A	29 mL
Percoll	42%	18.9 mL
**Total**	**N/A**	**50 mL**

Prepare in a sterile environment, aliquot 5 mL to polycarbonate tubes, and store at 4°C for up to six months.

**Table:** 44% Percoll

Reagent	Final concentration	Amount
10X PBS	N/A	2.2 mL
RPMI	N/A	28 mL
Percoll	44%	19.8 mL
**Total**	**N/A**	**50 mL**

Prepare in a sterile environment, aliquot 5 mL to polycarbonate tubes, and store at 4°C for up to six months.

**Table:** 70% Percoll

Reagent	Final concentration	Amount
10X PBS	N/A	3.5 mL
1X PBS	N/A	15 mL
Percoll	70%	31.5 mL
**Total**	**N/A**	**50 mL**

Prepare in a sterile environment, aliquot 3 mL to polycarbonate tubes, and store at 4°C for up to six months.

**CRITICAL:** Percoll should be stored in polycarbonate tubes to prevent silica particles adhering to the tube walls on prolonged storage, as indicated in the manufacturer’s instruction sheet (https://cdn.cytivalifesciences.com/api/public/content/digi-17134-pdf?_gl=1∗14xm7b2∗_gcl_au∗MTc2NzEwMDAyMy4xNzcwOTY5NDYz). Polypropylene tubes are suitable for use during centrifugation steps in this protocol.

### RBC lysis solution

Prepare as per the manufacturer’s instructions by diluting 1:10 in distilled water (https://cytek-web.s3.amazonaws.com/cytekbio.com/Tonbo/Protocols/protocol-lysing.pdf). Prepare fresh on the day of use and store at 21°C–23°C.

***Alternatives:*** RBC lysis solutions from other manufacturers can be used in this protocol following validation. Please refer to the Alternative Resources Table for additional details.

### Zombie UV viability dye

#### Stock

Prepare as per the manufacturer’s instructions (https://d1spbj2x7qk4bg.cloudfront.net/fr-fr/products/zombie-uv-fixable-viability-kit-9336?displayInline=true&filename=Zombie%20UV™%20Fixable%20Viability%20Kit.pdf&leftRightMargin=15&pdf=true&topBottomMargin=15&v=20260212073919). Briefly centrifuge the vial of lyophilized reagent and reconstitute using 100 μL of DMSO. Vortex to mix, then centrifuge. Store as 2 μL aliquots at −20°C, protected from light and discard after thawing.

**Table:** Working solution

Reagent	Final concentration	Amount
1X PBS	N/A	As required
Zombie UV	1:1000	As required
**Total**	**N/A**	**40 μL per stain**

Prepare fresh on the day of staining and store at 4°C protected from light.

**CRITICAL:** Zombie UV must be diluted in 1X PBS as Tris and protein-containing buffers can interfere with staining.

***Alternatives:*** Viability dyes conjugated to other fluorochromes or from other manufacturers can be used in this protocol following validation. Please refer to the Alternative Resources Table for additional details.

**Table:** Antibody cocktail

Reagent	Final concentration	Amount
Brilliant stain buffer	N/A	Up to 40 μL per stain
Surface antibodies	1:20 – 1:1000	As required
**Total**	**N/A**	**40 μL per stain**

Prepare fresh on the day of staining and store at 4°C protected from light.

**CRITICAL:** Before preparing the cocktail, centrifuge antibodies at 16,000 × *g* for 5 min at 4°C and take the required volume from the supernatant to avoid aggregates and debris in the final solution.

#### BD stabilizing fixative buffer

Stabilizing Fixative is recommended if samples are not being analyzed immediately to preserve cell morphology and reduce dye breakdown over time. Prepare as per the manufacturer’s instructions by diluting 1:3 in distilled water (https://www.bdbiosciences.com/content/dam/bdb/product_assets/product_pdf/instruments/pdf_0/23-8784.pdf). Prepare fresh on the day of staining and store at 21°C–23°C.

***Alternatives:*** BD Stabilizing Fixative is the strongly preferred fixative for use with tandem dye antibody conjugates. Other fixatives such as 1–2% paraformaldehyde in PBS can be used following validation, however the signal of some fluorochromes may be diminished. Please refer to the Alternative Resources Table for additional details.

#### BD LSRFortessa flow cytometer

The example antibody panel in this protocol was designed for compatibility with a 5-laser BD LSRFortessa. Adjustments to antibody selection and fluorochrome combinations may be required depending on the configuration of the available flow cytometer. Any panel containing a viability dye and CD8α marker can be used in this protocol. Additional antibodies may be included to identify contaminating cells.

**Table:** Alternative resources table

REAGENT or RESOURCE	ALTERNATIVE	SOURCE	IDENTIFIER
DMEM	RPMI-1640	Thermo Fisher	Cat#11875093
Red blood cell lysis solution	Red blood cell lysis solution Red blood cell lysis solution	BioLegend Miltenyi Biotec	Cat#420301 Cat#130-094-183
MACS buffer (MACS® BSA Stock Solution + autoMACS® Rinsing Solution)	RoboSep™ Buffer MojoSort™ Buffer	Stem Cell Technologies BioLegend	Cat#20104 Cat#480017
MS columns	LS columns	Miltenyi Biotec	Cat#130-042-401
MiniMACS™ Separator	OctoMACS™ Separator	Miltenyi Biotec	Cat#130-042-109
MidiMACS™ Separator	QuadroMACS™ Separator	Miltenyi Biotec	Cat#130-091-051
96-Well U bottom plates	5 mL Round bottom polystyrene tubes	Corning	Cat#352052
Zombie UV fixable viability kit	Fixable Viability Stain 450 Fixable Viability dye eFluor 455 UV GloCell Fixable Viability Dye UV450	BD Biosciences Thermo Fisher Stem Cell Technologies	Cat#562247 Cat#65–0868 Cat#75008.1
BD Stabilizing Fixative	Methanol-free Paraformaldehyde	ProSciTech ProSciTech	Cat#EMS15735-85 Cat#C004-100
Flowjo	Kaluza FCS Express CytoBank	Beckman Coulter De Novo Software Beckman Coulter	RRID:SCR_016182 RRID:SCR_016431 RRID:SCR_014043

The key resources table outlines the specific reagents and materials used to establish this protocol. The alternative resources listed above are anticipated to yield comparable results, yet any substitutions should undergo validation prior to their use.

## Step-by-step method details

### Tissue harvest

**Timing: 30 min per mouse**

Here, we describe harvesting of the liver, heart, and kidneys from mice, along with multiple bones (hindlimb, forelimb, sternum and spine) for subsequent BM processing.

1. Induce anesthesia and lay the mouse in a supine position, maintaining constant anesthetic delivery.
2. Tape the limbs outwards to permit access to the abdomen.
3. Sterilize the abdomen with 70% ethanol.
4. Open and perfuse the mouse:
  a. Lift the skin at the pelvis with forceps and create an incision using scissors.
  b. Continue the incision to the sternum to expose the peritoneal cavity membrane.
  c. Open the peritoneum by cutting from the pelvis to the sternum.
  d. Extend the opening by incising along both flanks.
  e. Gently move the abdominal organs aside with a cotton tip to reveal the abdominal aorta.
  f. Perfuse the mouse with 3 mL cold saline by inserting a 30 G needle attached to a 3 mL syringe into the abdominal aorta below the kidneys.
    ***Note:*** The abdominal organs should blanch and appear pale.
    i. During perfusion, cut the aorta.
    ii. Soak up the perfusate with a cotton ball.
5. Harvest the heart:
  a. Cut the lateral sides of the rib cage and diaphragm with scissors.
  b. Using clamp forceps, clamp and invert the sternum to expose the chest cavity.
  c. Resect the heart from the cavity, place in DMEM + 10% HI-NCS and keep on ice.
6. Resect the liver, place in DMEM and keep on ice.
7. Resect the kidneys, place in DMEM + 10% HI-NCS and keep on ice.
8. Resect the sternum, cutting away the ribs, place in DMEM and keep on ice.
9. Harvest the humerus and radiusc:
  a. Extend the midline incision from the shoulder across the forelimb.
  b. Using forceps, retract the posterior skin across the forelimb and remove it using scissors.
  c. Cut away all muscle tissue to expose the humerus and radius.
  d. Incise the connective tissue surrounding the humeral head to release the humerus.
  e. Dislocate the radius by rotating the paw at the wrist joint.
  f. Cut away any remaining muscle and tendons from the bones.
  g. Transfer bones to DMEM and keep on ice.
10. Harvest the femur and tibia:
  a. Extend the abdominal incision from the hip across the hindlimb.
  b. Using forceps, retract the posterior skin across the hindlimb and remove it using scissors.
  c. Cut away all muscle tissue to expose the femur and tibia.
  d. Incise the connective tissue surrounding the femoral head to release the femur.
  e. Dislocate the tibia by rotating the paw at the ankle joint.
  f. Cut away any remaining muscle and tendons from the bones.
  g. Transfer bones to DMEM and keep on ice.
11. Turn the mouse to a prone position and sterilize the back with 70% ethanol.
12. Harvest the spine:
  a. With forceps, lift the skin above the tail and make an incision using scissors.
  b. Continue the incision cranially to the base of the head to expose the spine.
  c. Use scissors to cut out the spine and remove all excess connective tissue.
  d. Cut the spine into smaller segments.
  e. Insert forceps into the openings of each segment and remove the spinal cord.
  f. Place spinal segments in DMEM and keep on ice.

***Note:*** Other tissues such as the spleen and lymph nodes can also be harvested for analysis.

### Preparation of single-cell suspension from liver tissue

**Timing: 50 min**

**Figure 1:**
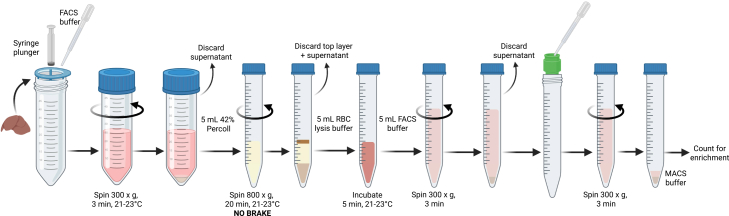
Illustration of liver leukocyte isolation procedure

This step outlines the mechanical dissociation and differential centrifugation of liver tissue to generate a single cell suspension of leukocytes for subsequent enrichment (Figure 1).

13. Place the liver in a 70 μm cell strainer atop a 50 mL tube.
14. Gently mash the liver through the strainer using the plunger of a 3 mL syringe, rinsing with FACS buffer.
15. Centrifuge at 300 × *g* for 3 min at 21–23°C, then carefully remove and discard the supernatant.
16. Resuspend the pellet in 1 mL of 42% Percoll and transfer to a 15 mL tube.
17. Rinse the original tube with an additional 4 mL of 42% Percoll and add this to the 15 mL tube.

**CRITICAL:** Percoll must be at 21°C–23°C before use.

18. Centrifuge at 800 × *g* for 20 min at 21°C–23°C with no brake.

**CRITICAL:** The brake must be off during centrifugation.

19. Carefully remove and discard the top cellular layer and Percoll from the pellet (leukocytes).
20. Resuspend the pellet in 100 μL of RBC Lysis buffer and transfer to a new 15 mL tube containing 4.9 mL of RBC Lysis buffer.

21. Use 100 μL of the suspension to wash the bottom of the original tube.
  ***Note:*** Avoid the contaminating non-leukocytes on the walls of the tube.
  a. Add this to the rest of the cell suspension and mix well.

22. Incubate for 5 min at 21–23°C.
23. Add 5 mL of FACS buffer and centrifuge at 300 × *g* for 3 min.

***Note:*** The resulting leukocyte pellet should now be pale.

24. Rinse a 20 μm pre-separation filter with 500 μL of FACS buffer and place atop a 15 mL tube.
25. Resuspend the pellet in FACS buffer and pass through the filter, rinsing with FACS buffer.
26. Centrifuge the suspension at 300 × *g* for 3 min, then carefully remove and discard the supernatant.
27. Resuspend the pellet in 100 μL of MACS buffer and use 5 μL of the cell suspension for counting (troubleshooting 2).

### Preparation of single-cell suspension from heart tissue

**Timing: 1 h**

**Figure 2:**
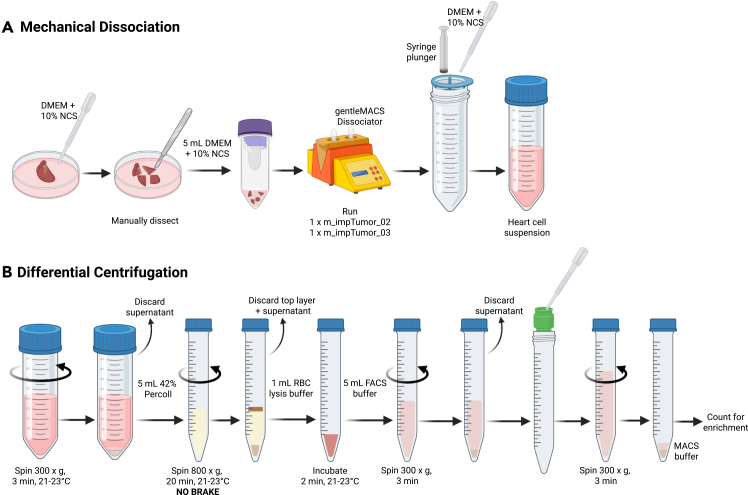
Illustration of heart leukocyte isolation procedure Leukocytes are isolated through (A) mechanical dissociation using the gentleMACS Dissociator followed by (B) density centrifugation using Percoll.

This step describes the process of tissue dissociation combined with differential centrifugation of heart tissue to generate a single cell suspension of leukocytes (Figure 2). Heart tissue is typically processed enzymatically to improve leukocyte viability and yield, however for downstream applications that require enzyme-free processing, mechanical dissociation is required. This protocol outlines a method for mechanical heart dissociation using the gentleMACS™ Dissociator, though protocols involving enzymatic digestion have been described.^1^^,^^2^

28. Transfer the heart to a 60 mm petri dish and cover with chilled DMEM + 10% HI-NCS.
29. Gently cut the heart into quarters with a scalpel and transfer to a C tube containing 5 mL of DMEM + 10% HI-NCS.
30. Tightly close the C Tube and attach it upside down onto the sleeve of the gentleMACS Dissociator.
  a. Run program m_impTumor_02.
  b. Run program m_impTumor_03.

***Optional:*** Briefly centrifuge the C tube to collect the heart tissue at the bottom of the tube.

31. Place a 70 μm cell strainer atop a 50 mL tube.
32. Gently mash the heart through the strainer using the plunger of a 3 mL syringe, rinsing with DMEM + 10% HI-NCS.
33. Centrifuge at 300 × *g* for 3 min at 21°C–23°C, then carefully remove and discard the supernatant.
34. Resuspend the cell pellet in 1 mL of 42% Percoll and transfer to a 15 mL tube.
35. Rinse the original tube with an additional 4 mL of 42% Percoll and add this to the 15 mL tube.

**CRITICAL:** Percoll must be at 21°C–23°C before use.

36. Centrifuge at 800 x g for 20 min at 21°C–23°C with no brake.

**CRITICAL:** The brake must be off during centrifugation.

37. Carefully remove and discard the top cellular layer and Percoll from the pellet (leukocytes).
38. Resuspend the pellet in 100 μL of RBC Lysis buffer and transfer to a new 15 mL tube containing 900 μL of RBC Lysis buffer.

39. Use 100 μL of the suspension to wash the bottom of the original tube.
  ***Note:*** Avoid the contaminating non-leukocytes on the walls of the tube.
  a. Add this to the rest of the cell suspension and mix well.

40. Incubate for 2 min at 21°C–23°C.
41. Add 5 mL of FACS buffer and centrifuge at 300 × *g* for 3 min.

***Note:*** The resulting leukocyte pellet should now be pale.

42. Rinse a 20 μm pre-separation filter with 500 μL of FACS buffer and place atop a 15 mL tube.
43. Resuspend the pellet in FACS buffer and pass through the filter, rinsing with FACS buffer.
44. Centrifuge the suspension at 300 x g for 3 min, then carefully remove and discard the supernatant.
45. Resuspend the pellet in 100 μL of MACS buffer and use 5 μL of the cell suspension for counting (troubleshooting 2).

### Preparation of single-cell suspension from kidney tissue

**Timing: 1 h**

**Figure 3:**
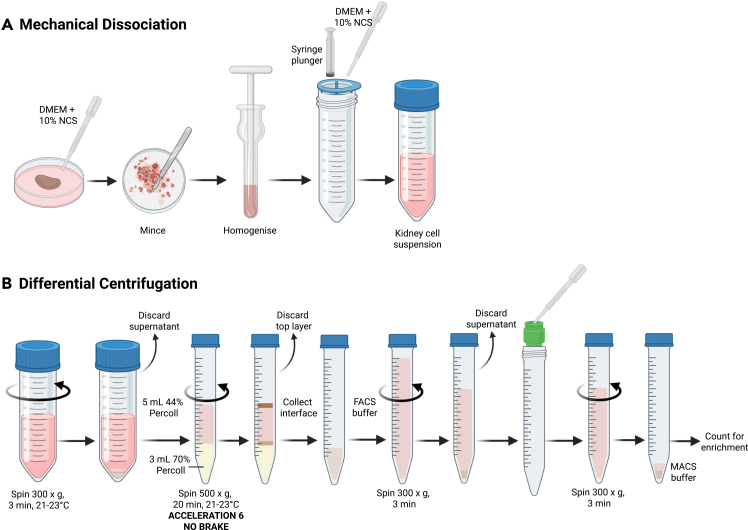
Illustration of kidney leukocyte isolation procedure Leukocytes are isolated through (A) mechanical dissociation using a dounce homogenizer followed by (B) density centrifugation using Percoll.

This step describes the process of tissue dissociation combined with differential centrifugation of kidney tissue to generate a single cell suspension of leukocytes (Figure 3). As for the heart, kidney tissue can be enzymatically or mechanically dissociated. This protocol outlines a method for mechanical kidney dissociation using a dounce homogenizer, though protocols involving enzymatic digestion have been described.^3^^,^^4^

46. Transfer the kidney to a 60 mm petri dish and cover with DMEM + 10% HI-NCS.
47. Gently cut the kidney into ∼3mm pieces using a scalpel.
48. Transfer the kidney into a pre-chilled dounce homogenizer containing ∼5 mL chilled DMEM + 10% HI-NCS.
49. Working on ice, homogenize the tissue by twisting and re-inserting the long end of the T-shaped pestle into the collection piece of the dounce homogenizer until there are no visible tissue pieces left.

**CRITICAL:** Keep the pestle below the surface of the tissue suspension to prevent bubble formation, which can obscure assessment of homogenization.

***Note:*** The volume of DMEM added to the dounce homogenizer will depend on the capacity of the chamber. Exceeding one third of the chamber volume may lead to spillage.

50. Place a 70 μm cell strainer atop a 50 mL tube.
51. Gently mash the kidney through the strainer using the plunger of a 3 mL syringe, rinsing with FACS buffer.
52. Centrifuge at 300 x g for 3 min at 21°C–23°C, then carefully remove and discard the supernatant.
53. Resuspend the pellet in 5 mL 44% Percoll. Transfer 3 mL of 70% Percoll into a 15 mL tube.

**CRITICAL:** Percoll must be at 21°C–23°C before use.

54. Slowly overlay the kidney-44% Percoll mix on top of the 70% Percoll in the 15 mL tube.
55. Centrifuge at 500 × *g* for 20 min at 21°C–23°C using acceleration 6 and no brake.

**CRITICAL:** The brake must be off during centrifugation.

56. Carefully remove and discard the top cellular layer.
57. Collect the interface (leukocytes) of the two Percoll layers and transfer to a new 15 mL tube.
58. Top up to 15 mL with FACS buffer, invert to mix, and centrifuge at 300 × *g* for 3 min to pellet the leukocytes.
59. Rinse a 20 μm pre-separation filter with 500 μL of FACS buffer and place atop a 15 mL tube.
60. Resuspend the pellet in FACS buffer and pass through the filter, rinsing with FACS buffer.
61. Centrifuge the suspension at 300 × *g* for 3 min, then carefully remove and discard the supernatant.
62. Resuspend the pellet in 100 μL of MACS buffer and use 5 μL of the cell suspension for counting (troubleshooting 2).

### Preparation of single-cell suspension from BM

**Timing: 50 min**

**Figure 4:**
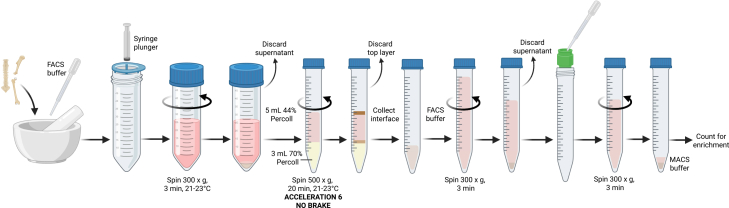
Illustration of bone marrow leukocyte isolation procedure

This step outlines the mechanical disruption of whole bones to generate a single cell suspension of BM leukocytes (Figure 4). Alternatively, BM can be flushed from long bones using a syringe, though this method will greatly reduce cell yield.

63. Place a 70 μm cell strainer atop a 50 mL tube.
64. Place bones into a mortar and add ∼1 mL FACS buffer per bone.
65. Crush each bone by pressing down firmly with a pestle, until the bone becomes clear and the BM has been released.
66. Pass the liquid through the 70 μm cell strainer. Mash the BM with the plunger of a 3 mL syringe, rinsing with FACS buffer.
67. Rinse the bones, mortar and pestle with FACS buffer, and pass this through the same strainer.
68. Centrifuge the cell suspension at 300 × *g* for 3 min at 21°C–23°C then carefully remove and discard the supernatant.
69. Resuspend the pellet in 5 mL 44% Percoll. Transfer 3 mL of 70% Percoll into a 15 mL tube.

**CRITICAL:** Percoll must be at 21°C–23°C before use.

70. Slowly overlay the BM-44% Percoll mix on top of the 70% Percoll in the 15 mL tube.
71. Centrifuge at 500 × *g* for 20 min at 21°C–23°C using acceleration 6 and no brake.

**CRITICAL:** The brake must be off during centrifugation.

72. Carefully remove and discard the top cellular layer.
73. Collect the interface (leukocytes) of the two Percoll layers and transfer to a new 15 mL tube.
74. Top up to 15 mL with FACS buffer, invert to mix, and centrifuge at 300 × *g* for 3 min to pellet the leukocytes.
75. Rinse a 20 μm pre-separation filter with 500 μL of FACS buffer and place atop a 15 mL tube.
76. Resuspend the pellet in FACS buffer and pass through the filter, rinsing with FACS buffer. Carefully scrape the bottom of the filter with a transfer pipette if the suspension becomes blocked (troubleshooting 1).

**CRITICAL:** Do not substitute the 20 μm pre-separation filter with a larger cell strainer (e.g. 40 μm), as bone fragments can traverse the filter and interfere with downstream enrichment.

77. Centrifuge the suspension at 300 × *g* for 3 min, then carefully remove and discard the supernatant.
78. Resuspend the pellet in ∼6 mL of MACS buffer and use 5 μL of the cell suspension for counting (troubleshooting 2).

**CRITICAL:** Do not perform RBC lysis on this tissue as doing so will reduce the purity of downstream enrichment.

### CD8 T cell enrichment

**Timing: 35 min per sample**

**Figure 5:**
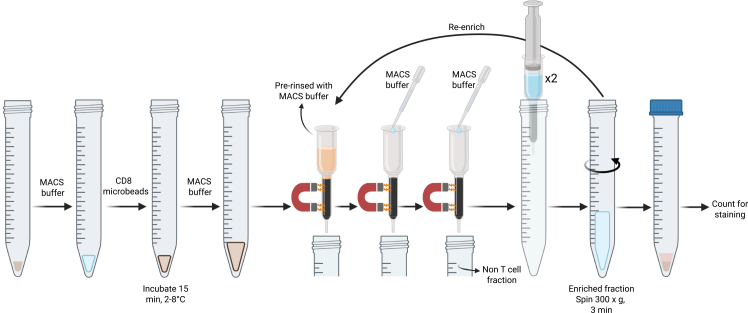
Illustration of CD8 T cell enrichment process

Here, we describe the enrichment of CD8 T cells from single cell suspensions of liver, heart, kidney and BM cells using the Miltenyi Biotec CD8 (TIL) MicroBeads (Figure 5), which were developed for the isolation of CD8 T cells from mouse tumors. We found that these microbeads are required over regular CD8a (Ly-2) microbeads due to the considerable amount of debris present in processed liver, heart, kidney and BM samples, similar tumors. Both MS and LS MACS columns can be used with MACS magnetic separators, with differences summarized in Table 1. MS columns were used in this protocol to maximize post-enrichment purity; use of LS columns would require validation.

***Note:*** An aliquot of the original sample may be collected prior to enrichment and later stained with the enriched fraction for calculation of CD8 T cell recovery.

79. Transfer 1 × 10^7^ to 5 × 10^7^ cells to a new 15 mL tube.
80. Centrifuge the cell suspension at 300 × *g* for 3 min and aspirate the supernatant.
81. Resuspend the cell pellet in 90 μL chilled MACS buffer per 10^7^ cells.

**CRITICAL:** When working with fewer than 10^7^ cells, use the same volumes as indicated for 1 × 10^7^ cells. Do not scale down. For greater cell numbers, scale up reagent and buffer volumes accordingly.

**CRITICAL:** Always use freshly prepared MACS buffer and keep cold (2–8°C).

82. Add 10 μL of CD8 (TIL) MicroBeads per 10^7^ cells.
83. Mix well and incubate for 15 min at 2–8°C.
84. Add MACS buffer to a final volume of 500 μL for up to 2 × 10^7^ cells (MS columns) or 5 × 10^7^ cells (LS columns).

***Note:*** For greater cell numbers, samples can be distributed over several columns during separation.

85. Insert column into the magnet of a compatible MACS separator.
86. Rinse column with the appropriate volume of MACS buffer.
  a. MS columns: 500 μL.
  b. LS columns: 3 mL.

**CRITICAL:** Wait until the column reservoir is completely empty between steps.

**CRITICAL:** Avoid pipetting bubbles into the column reservoir to prevent blockages (troubleshooting 3).

87. Apply the cell suspension onto the column. Collect the flow-through into a 15 mL tube.
88. Wash column with the appropriate volume of MACS buffer.
  a. MS columns: 500 μL.
  b. LS columns: 1 mL.
89. Wash column a second time with the appropriate volume of MACS buffer.
  a. MS columns: 500 μL.
  b. LS columns: 1 mL.
90. Remove column from the magnet and place it onto a new 15 mL tube.
91. Pipette the appropriate volume of MACS buffer into the column. Press the plunger firmly and smoothly into the column to flush out the magnetically labeled cells.
  a. MS columns: 1 mL.
  b. LS columns: 3 mL.

***Optional:*** To increase cell recovery, repeat step 91, flushing the column again.

**Table 1:** MS vs LS MACS columns

Column	Maximum no. of labeled cells	Maximum no. of total cells	Compatible separators
MS	1 × 10^7^	2 × 10^7^	MiniMACS, OctoMACS, VarioMACS, SuperMACS II
LS	4 × 10^7^	5 × 10^7^	MidiMACS, QuadroMACS, VarioMACS, SuperMACS II, MultiMACS Cell24 Separator Plus

To increase purity, the eluted fraction can be enriched over a new column:

92. Repeat steps 85–91 using a new column and the eluted fraction from step 91.
93. Pellet the cells by centrifugation at 300 × *g* for 3 min and discard the supernatant.
94. Continue with the ‘staining for flow cytometry’ step below to assess viability and purity (troubleshooting 4).

### Staining for flow cytometry

**Timing: 1 h 20 min**

This step details the method for staining the enriched sample to assess CD8 T cell purity and viability. An aliquot of the original sample may also be stained. Here we describe staining using a 96 well U-bottom plate, though individual FACS tubes may also be used.

95. Transfer 0.1–4 × 10^6^ cells per well.
  ***Note:*** This protocol uses Zombie UV dye as a viability stain, prior to surface antibody staining. Zombie UV is incompatible with protein-containing buffers, so cells are first washed in PBS.
96. Centrifuge at 200 × *g* for 3 min at 21°C–23°C and discard supernatant.
97. Wash the cells by resuspending in 150 μL PBS, then centrifuge at 200 × *g* for 3 min at 21°C–23°C and discard supernatant.
98. Resuspend the cells in 40 μL Zombie UV diluted 1:1000 in PBS.
99. Incubate in the dark for 20 min at 21°C–23°C.
100. Add 150 μL FACS buffer then pellet the cells by centrifugation at 200 × *g* for 3 min at 21°C–23°C and discard supernatant.
  ***Note:*** Mouse CD8 T cells are primarily identified by the expression of the CD8α (Ly-2) protein. A CD8α marker is required for identification of CD8 T cells by flow cytometry. Inclusion of other leukocyte markers may be useful to identify potentially contaminating cells. An example panel is provided in Table 2, with the corresponding gating strategy shown in Figure S1.
101. Resuspend the cells in 40 μL surface antibody cocktail as per Table 2.

**Table 2:** Example flow cytometry panel to analyze major leukocyte subsets on a 5 laser BD LSRFortessa flow cytometer

Laser	Detector	Fluorophore	Antigen	Dilution
355	379_28	BUV395	CD11b	1:200
355	450_50	Zombie UV	Live/dead	1:1000
355	740_35	BUV737	NKp46	1:100
405	525_50	BV480	CD8β	1:800
405	610_20	SB600	CD4	1:300
405	670_30	SB645	CD8α	1:200
488	530_30	FITC	Ly6G	1:100
561	610_20	AF594	B220	1:400
640	670_30	AF647	F4/80	1:100
640	730_45	AF700	TCRβ	1:50
640	780_60	APCeF780	CD45.2	1:200

See also Figure S1.
102. Incubate in the dark for 20 min at 21°C–23°C.
103. Add 150 μL FACS buffer then pellet the cells by centrifugation at 200 × *g* for 3 min at 21°C–23°C and discard supernatant.
104. Wash the cells with 150 μL FACS buffer.
105. Resuspend the cells in 100 μL 1× BD Stabilizing Fixative.
106. Incubate in the dark for 15 min at 21°C–23°C.
107. Add 100 μL FACS buffer then pellet the cells by centrifugation at 200 × *g* for 3 min at 21°C–23°C and discard supernatant.
108. Resuspend in 100 μL FACS buffer.
109. Transfer samples to a tube or plate appropriate for acquisition on the available flow cytometer (troubleshooting 5).

**Pause point:** Prior to analysis, cells may be stored for up to 4 days at 4°C, protected from light.

## Expected outcomes

In cell separation, there is an inherent trade-off between purity (the percentage of desired cells in the enriched sample) and yield (the number of desired cells recovered). This protocol has been optimized to obtain the highest tissue-specific CD8 T cell purities. However, variables such as those listed in Table 3 may be adjusted to alter the balance between purity and yield, depending on the downstream application.

**Table 3:** Variables influencing post-enrichment cell purity and yield

Variable	Purity	Yield
Increased number of column separations	↑	↓
Increased number of flushes	↓	↑
Use of MS columns^a^	↓	↑
Use of LS columns^a^	↑	↓
Improved sample quality/viability	↑	↑

a As indicated by the manufacturer.

**Figure 6:**
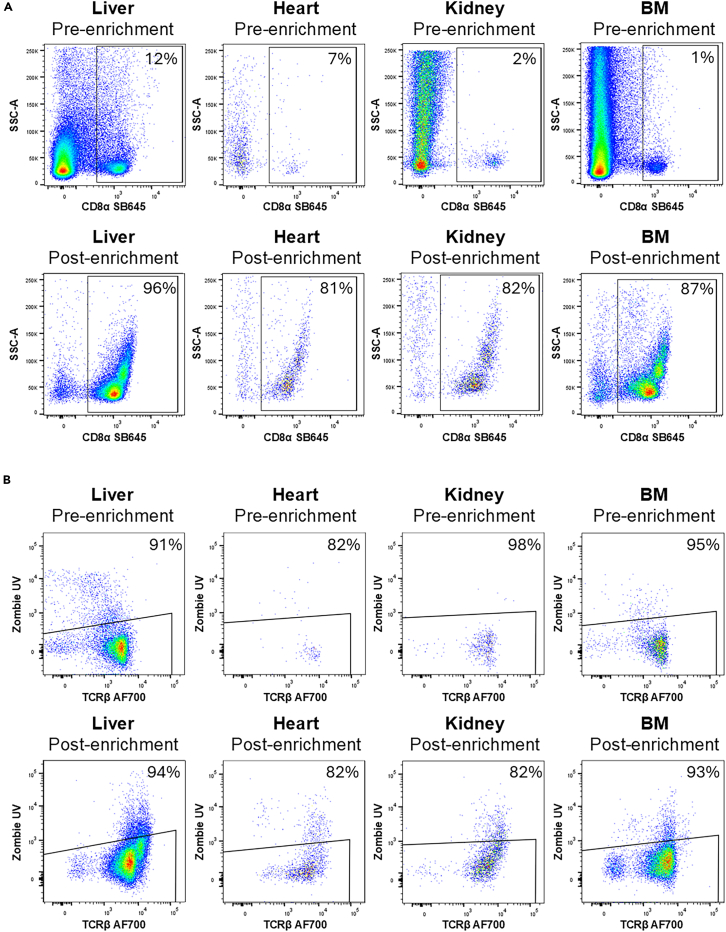
Flow cytometry enrichment analysis FACS plots showing (A) example CD8 T cell purity and (B) example CD8 T cell viability of pre- and post-enriched liver, heart, kidney and BM samples.

Expected purity and viability of enriched CD8 T cells are shown in Figure 6. We have found purity to be consistent across each tissue type: liver (90–96%), heart (74–81%), kidney (70–82%), and BM (81–87%) from n=3 separate experiments for each tissue type. As shown, the starting frequency of CD8 T cells differs across tissues, which contributes to the variation in purities obtained following enrichment. The expected total cell numbers following tissue processing to a single cell suspension, along with post-enrichment CD8 T cell yields are summarized in Table 4. These values were obtained using tissues from naïve mice and are provided as a guide only, as values will vary depending on the experimental model used.

**Table 4:** Expected leukocyte count and CD8 T cell yield following tissue processing and enrichment

Tissue	Total number of leukocytes (x10^6^)	Viability of leukocytes (%)	CD8 T cell yield (%)	Number of CD8 T cells enriched (x10^4^)	Viability of enriched CD8 T cells (%)
Liver	4.1 (2.6–6.9)	94 (93–97)	32 (11–42)	12 (9.5–16)	94 (94–96)
Heart^a^	0.07 (0.06–0.08)	91 (89–93)	76 (61–93)	0.48 (0.38–0.63)	78 (71–82)
Kidney^a^	1.1 (0.22–2.4)	90 (83–95)	5.5 (2.3–7.7)	0.35 (0.32–0.39)	84 (81–88)
BM^b^	132 (102–202)	97 (97–98)	28 (21–32)	6.1 (5.3–6.9)	89 (86–95)

Data shown is mean with range from n=3 experiments per tissue.

a Heart and kidney data were obtained from pooled organs and normalized to a single heart or kidney.

b BM data represents cells pooled from the sternum, spine, and two hind legs.

## Quantification and statistical analysis

Cell isolation efficiency can be determined through assessment of CD8 T cell recovery (yield) and purity. This requires aliquots of both pre- and post-enrichment samples to be analyzed by flow cytometry. Yield is calculated using the following formula:

$$\%\;\text{Yield}=\frac{\left[\left(TNC\;in\;enriched\;fraction\right)\times \left({\%\;purity\;of\;enriched\;fraction}^{**}\right)\right]}{\left[\left(TNC\;in\;original\;sample\right)\times \left({starting\;\%\;of\;desired\;cells}^{**}\right)\right]}\times 100$$

∗TNC = total number of cells.

∗∗As determined by flow cytometry.

Viability may be determined through cell counting and/or by flow cytometry. Manual or automated counting is recommended if viability is important for downstream processing.

## Limitations

The results presented here were obtained from tissues of naïve mice only. Magnetic separation efficiency can be compromised by high levels of cell death and debris, which may occur under certain experimental conditions. Efficiency may also be reduced when starting cell numbers are low. For heart and kidney samples, we pooled at least 2–3 organs to obtain sufficient cells for enrichment, although this may not be feasible in all studies. Initial cell numbers and potential yields will vary depending on the experimental model (e.g., naïve vs tumor-bearing mice) and biological factors such as mouse strain, sex, age, and weight, which influence baseline immune cell frequencies. The yield required ultimately depends on the downstream application.

We have found that positive selection yields superior recovery of CD8 T cells compared to negative selection, likely due to their relatively low frequency in the liver, heart, kidney, and BM. However, following positive selection, the enriched cells remain bound by conjugated antibodies and beads which may interfere with certain downstream applications. Commercial kits enabling the release of beads from cells after enrichment are available but would require testing.

The described enrichment protocol involves multiple washing, centrifugation and filtration steps, each of which introduces an opportunity for cell loss, cumulatively reducing T cell yield. Filtration through 20 μm strainers, whilst essential for debris removal, may also retain larger immune cell subsets (e.g., activated lymphocytes), contributing to unintended cell loss. Immunomagnetic separation also has limited scalability. Unless an automated cell separator is acquired, steps such as manual pipetting make it challenging to process large numbers of samples in parallel, becoming unfeasible for large-scale studies.

## Troubleshooting

### Problem 1

Unable to pass BM through separation filter during preparation of a single cell suspension (related to step 76).

### Potential solution

**Figure 7:**
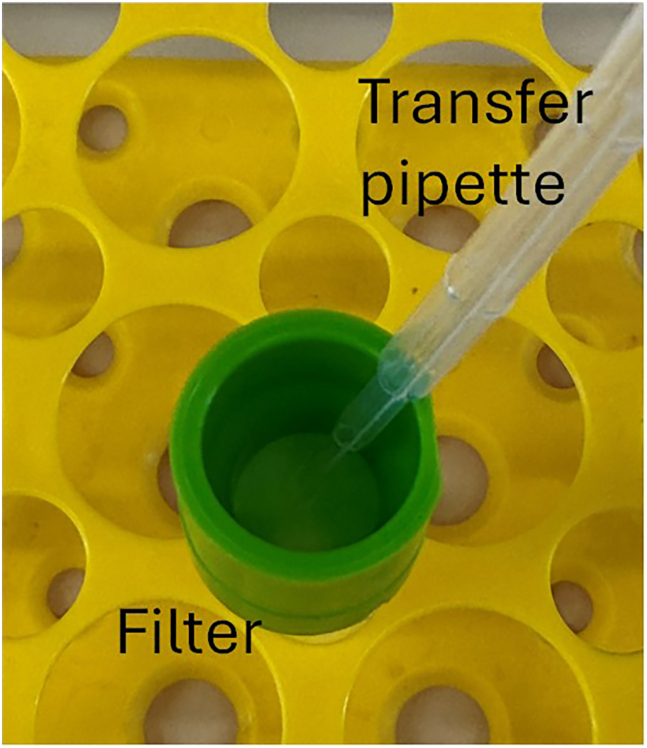
Example clearing of 20 μm pre-separation filter

Crushing whole bones to release BM introduces bone fragments that may cause difficulty in passing the suspension through a 20 μm separation filter. The fragments build up on the small surface area of the filter, causing a blockage. To rectify this issue, a transfer pipette can be used to gently scrape the filter surface, moving aside trapped contents so that the suspension can pass through (Figure 7).

### Problem 2

Low leukocyte yield or poor viability pre-enrichment (related to step 27, step 45, step 62, and step 78).

### Potential solution

Low leukocyte recovery and/or viability can arise from sample degradation or cell loss during processing. Tissue must be kept moist throughout the procedure, including during mincing and passage through filters, as drying will reduce viability. Samples should also be kept on ice after harvest and between processing steps, except during incubations performed at 21°C–23°C. Preparing reagents and equipment in advance will further reduce sample time at suboptimal conditions. To minimize sample loss, all strainers should be thoroughly rinsed on both sides after passing the tissue or cell suspension through.

### Problem 3

Blocked column during enrichment (related to step 86).

### Potential solution

Suboptimal sample preparation, introduction of air bubbles and exceeding column capacity can all result in a blocked column during enrichment. Use of 20 μm pre-separation filters are critical to prevent tissue debris or clumps from physically blocking the column. To prevent blockage from air bubbles, MACS buffer may be degassed prior to use. If bubbles do form they should be removed by gently agitating the solution. If cell numbers exceed the maximum column capacity, buffer volumes should be increased proportionally and divided across multiple columns to prevent overloading.

### Problem 4

Low CD8 T cell yield or poor purity post-enrichment (related to step 94).

### Potential solution

Low CD8 T cell recovery and/or purity in the enriched fraction can result from errors occurring before or during the enrichment procedure. The original sample must be carefully counted, as inaccurate starting counts will compromise enrichment efficiency. Cell viability should also be maximized prior to enrichment, as dead cells can bind antibodies non-specifically and lead to reduced purity. During enrichment, the cell suspension should be pipetted onto the bottom and not the sides of the column reservoir, to prevent undesired cells from being retained in the column. Drying of the column can also result in impaired binding efficiency, hence the cell suspension and subsequent washes should be applied immediately once the reservoir empties. All buffers must be kept chilled, as use of warm buffers or substitution with non-recommended media will impede separation. Finally, knowledge of flow cytometry analysis is required for accurate assessment of purity.

### Problem 5

Poor or inaccurate resolution of positive CD8 populations following flow cytometry staining (related to step 109).

### Potential solution

Compromised reagents, background fluorescence, aggregate contamination, or inadequate panel optimization may result in misidentification or poor delineation of CD8 T cells on FACS plots. Fluorescent reagents are light-sensitive and should be protected from light during storage, cocktail preparation, and staining to prevent damage. Antibodies should be centrifuged before use to remove aggregates, and adequate washes should be performed after staining to remove excess, unbound dye. Antibody dilutions provided in this protocol serve as a starting point, but antibodies should be titrated for specific flow cytometers to achieve clear separation between negative and positive populations while minimizing fluorescence spillover into adjacent detectors. Fluorescence minus one controls may also be used to accurately define gating boundaries.

## Resource availability

### Lead contact

Further information and requests for resources and reagents should be directed to and will be fulfilled by the lead contact, Amy Prosser (amy.prosser@uwa.edu.au).

### Technical contact

Technical questions on executing this protocol should be directed to and will be answered by the technical contact, Ella Trolio (ella.trolio@uwa.edu.au).

### Materials availability

This study did not generate new unique reagents.

### Data and code availability

This study did not generate or analyze datasets or code.

## Acknowledgments

This work was supported by the 10.13039/501100001063Raine Medical Research Foundation (RPG070-2024 and RCA008-2024), The 10.13039/501100001801University of Western Australia (2024/GR001408), the Charlies Foundation for Research (DPG24-25_05), and the Western Australian Department of Health. The authors acknowledge the facilities and assistance of the Centre for Microscopy, Characterisation and Analysis, The 10.13039/501100001801University of Western Australia, a facility funded by the University, State, and Commonwealth governments. The authors also thank the scientific support teams of Miltenyi Biotec and STEMCELL Technologies and A/Prof. Alec Redwood for their technical guidance and Dr. Stephanie Huang, Mrs. Liu Liu, Mrs. Jaskirat Kaur, Dr. Omar Elaskalani, and Ms. Alexandra Monson for their assistance. The graphical abstract and figures were created using BioRender.com.

## Author contributions

Conceptualization, E.T. and A.P.; methodology, E.T., L.D., and A.P.; investigation, E.T., L.D., and A.P.; data curation, E.T.; writing – original draft, E.T.; writing – review and editing, E.T. and A.P.; funding acquisition, A.P. and M.L.

## Declaration of interests

The authors declare no competing interests.
