# Supplementary material for: Protocol for immunomagnetic enrichment of CD8 T cells from complex murine tissues
Source: STAR Protoc. 2026 Apr 2;7(2):104456. doi: 10.1016/j.xpro.2026.104456 (PMC13084401; doi:10.1016/j.xpro.2026.104456)
Supplement: Document S1. Figure S1 [file mmc1.pdf]

Supplemental Information

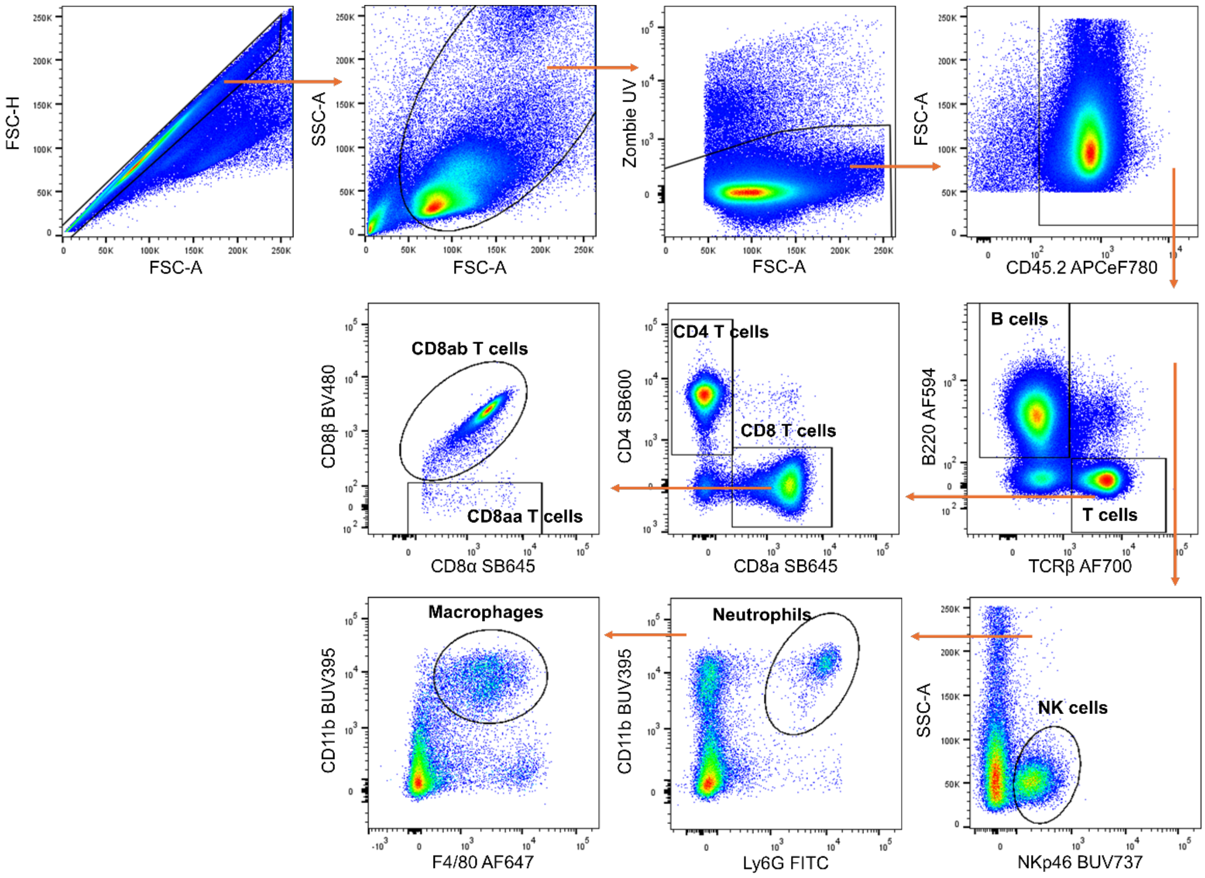

Figure S1. Flow cytometry gating strategy, related to Table 2. Example gating strategy to identify T cells, B cells, natural killer (NK) cells and myeloid cells (macrophages/neutrophils) in unenriched mouse tissue.
